# Regulatory mechanisms leading to differential Acyl-CoA synthetase 4 expression in breast cancer cells

**DOI:** 10.1038/s41598-019-46776-7

**Published:** 2019-07-16

**Authors:** Melina A. Dattilo, Yanina Benzo, Lucía M. Herrera, Jesica G. Prada, Ana F. Castillo, Ulises D. Orlando, Ernesto J. Podesta, Paula M. Maloberti

**Affiliations:** Biomedical Research Institute, INBIOMED, Department of Biochemistry, School of Medicine, University of Buenos Aires, CABA, Buenos Aires, Argentina

**Keywords:** Breast cancer, Transcriptional regulatory elements

## Abstract

Acyl-CoA synthetase 4 (ACSL4) overexpression plays a causal role in the aggressiveness of triple negative breast cancer. In turn, a negative correlation has been established between ACSL4 and estrogen receptor alpha (ERα) expression. However, the upstream regulatory mechanisms leading to differential ACSL4 expression between triple negative breast cancer and ERα-positive cells remained unknown. We performed the characterization of the human ACSL4 promoter and the identification of transcription factors involved. Deletional analysis demonstrated the proximal 43 base pairs of the promoter are involved in overexpression. By site directed mutagenesis we describe that retinoid-related orphan receptor alpha (RORα), Sp1 and E2F elements are involved in the promoter activity. We established for the first time that estrogen-related receptor alpha (ERRα) is a transcription factor involved in the higher activation of the human ACSL4 promoter in breast cancer cells. Furthermore, a combination of inhibitors of ACSL4 and ERRα produced a synergistic decrease in MDA-MB-231 cell proliferation. We also demonstrated that ERα restoration in triple negative breast cancer cells downregulates ACSL4 expression. The results presented in this manuscript demonstrated transcriptional mechanism is involved in the different expression of ACSL4 in human breast cancer cell lines of different aggressiveness.

## Introduction

It has been demonstrated that Acyl-CoA synthetase 4 (ACSL4) enzyme expression is elevated in cancer cells, which promotes an aggressive phenotype associated with the dysregulated production of eicosanoids, particularly in breast, colon, hepatocellular and prostate cancer^1–8^. Previous reports by our group describing the role of ACSL4 in the aggressiveness of breast cancer^1–3^ have shown high expression and mRNA levels of this enzyme in highly aggressive triple negative breast cancer (TNBC) cell lines, which lack estrogen receptor alpha (ERα) and progesterone receptor and do not overexpress human epidermal growth factor 2 receptor protein, as compared to ERα-expressing cells of lower aggressiveness. Moreover, reports by other authors have shown a correlation between ACSL4 expression and aggressiveness in TNBC tumors in patients^5,9^.

Our group has further established a causal role of ACSL4 expression in the transformation of a non-aggressive phenotype into a highly aggressive one *in vitro* and *in vivo*^1,3^. Regarding functional aspects, our group and others have established ACSL4 involvement in the mechanism underlying increased breast cancer cell proliferation, invasion and migration, *in vitro* and *in vivo*^1,3,5,9^.

Using RNA-seq and functional proteomics, we have reported that the single overexpression of ACSL4 in breast cancer cells of low aggressiveness regulates a broad spectrum of signaling pathways involved in both tumorigenesis and resistance to conventional treatments^2^. ACSL4 regulates signal transduction pathways implicated in cancer, such as the mTOR pathway, and its overexpression has been shown to decrease ERα levels^2^. In addition, we have shown the participation of ACSL4 in tumor resistance to hormone therapy^2^ and chemotherapy through the action of ABC transporters, with its downregulation or inhibition in TNBC cells contributing to effective therapeutic approaches^10^. Worth pointing out, while these studies focused on analyzing downstream elements regulated by ACSL4, the cellular mechanisms regulating ACSL4 expression under physiological and pathological conditions remain mostly unknown.

In particular, although the role of ACSL4 mediating an aggressive phenotype in breast cancer is well accepted, the regulation mechanisms involved in its overexpression in TNBC have not been elucidated yet. In this scenario, the current work analyzes the mechanisms underlying the differential regulation of ACSL4 in TNBC and ERα-positive breast cancer cells, which may allow to identify therapeutic targets and possible future drug combinations. Our studies have rendered a successful characterization of the human *ACSL4* promoter and different mechanisms regulating its action in breast cancer cells. Moreover, we demonstrate that estrogen-related receptor alpha (ERRα) is involved in the transcriptional upregulation of the ACSL4 gene.

## Results

### Functional characterization of *ACSL4* promoter in human breast cancer cell lines

**Figure 1:**
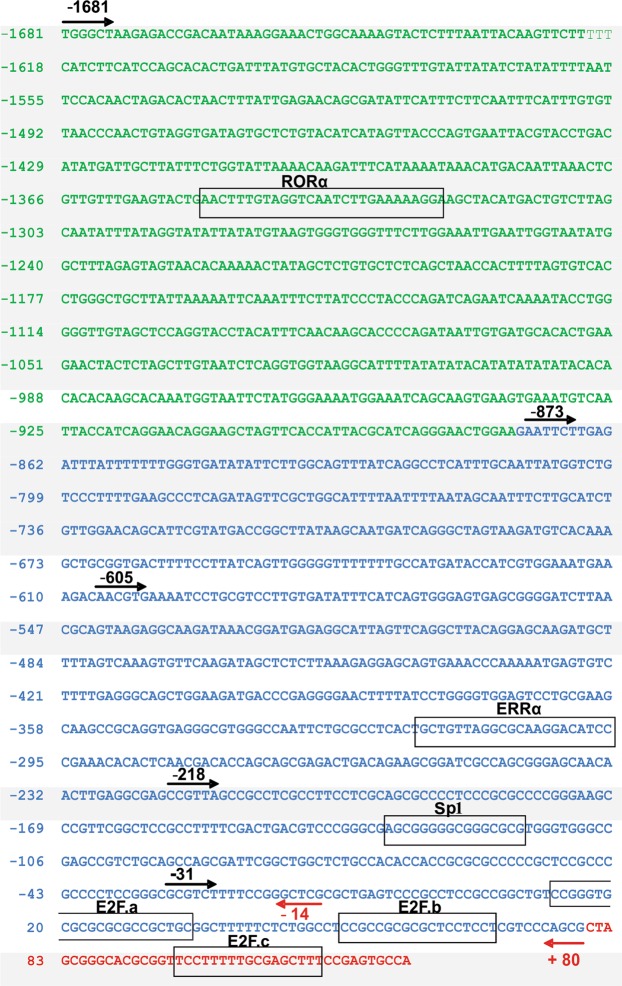
Nucleotide sequence of the human *ACSL4* promoter and a part of exon 1. Sequences from −1681 to −874 (green), −873 to +80 (blue), and +81 to +123 (red). Black arrows indicate the positions of 5′ seriated deletions. Red arrows indicate the positions of 3′ seriated deletions. Putative transcription factor binding sites are boxed.

**Figure 2:**
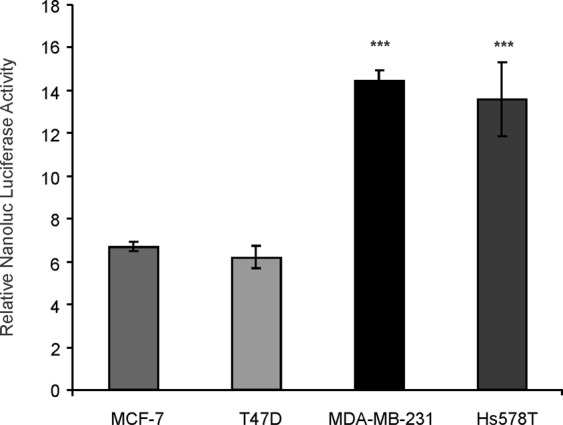
*ACSL4* promoter activity in breast cancer cell lines. The *ACSL4* human promoter sequence and part of exon 1, from positions −1681 to +123, was cloned into the pNL1.1 vector upstream of the Nanoluc Luciferase gene (*Nluc*). This construct was then transfected into MCF-7, T47D, MDA-MB-231 and Hs578T cell lines. Cells were allowed to recover for 48 h, and Nanoluc Luciferase activity was then determined by luminescence. Nanoluc luciferase activity was normalized to EGFP fluorescence counts measured in the same equipment and expressed as arbitrary units as described in Material and Methods. For comparison between lines, the promoter activity was relativized to the mean of the luminescence units of the empty pNL1.1 vector. Results are expressed as the mean of +/−SEM arbitrary units of at least three independent experiments. ***p < 0.001 *vs* MCF-7.

The construction pNL1.1-1681 carrying a fragment of ~1.8 kb in length of the human *ACSL4* promoter which contains also most of the exon 1 sequence (Fig. 1) was analyzed by transient transfection in breast cancer cell lines (Fig. 2). The functionality of the human *ACSL4* promoter in the regulation of transcription was assessed by its ability to drive the expression of the NanoLuc luciferase gene. The promoter was active in MDA-MB-231, T47D, Hs578T and MCF-7 cell lines. Most interestingly, the magnitude of activity in ERα-positive cells was significantly lower than in TNBC cells, about half in value (Fig. 2). This transcriptional ability of the *ACSL4* promoter correlates with previous observations of differences between lines in ACSL4 expression^1,9^.

**Figure 3:**
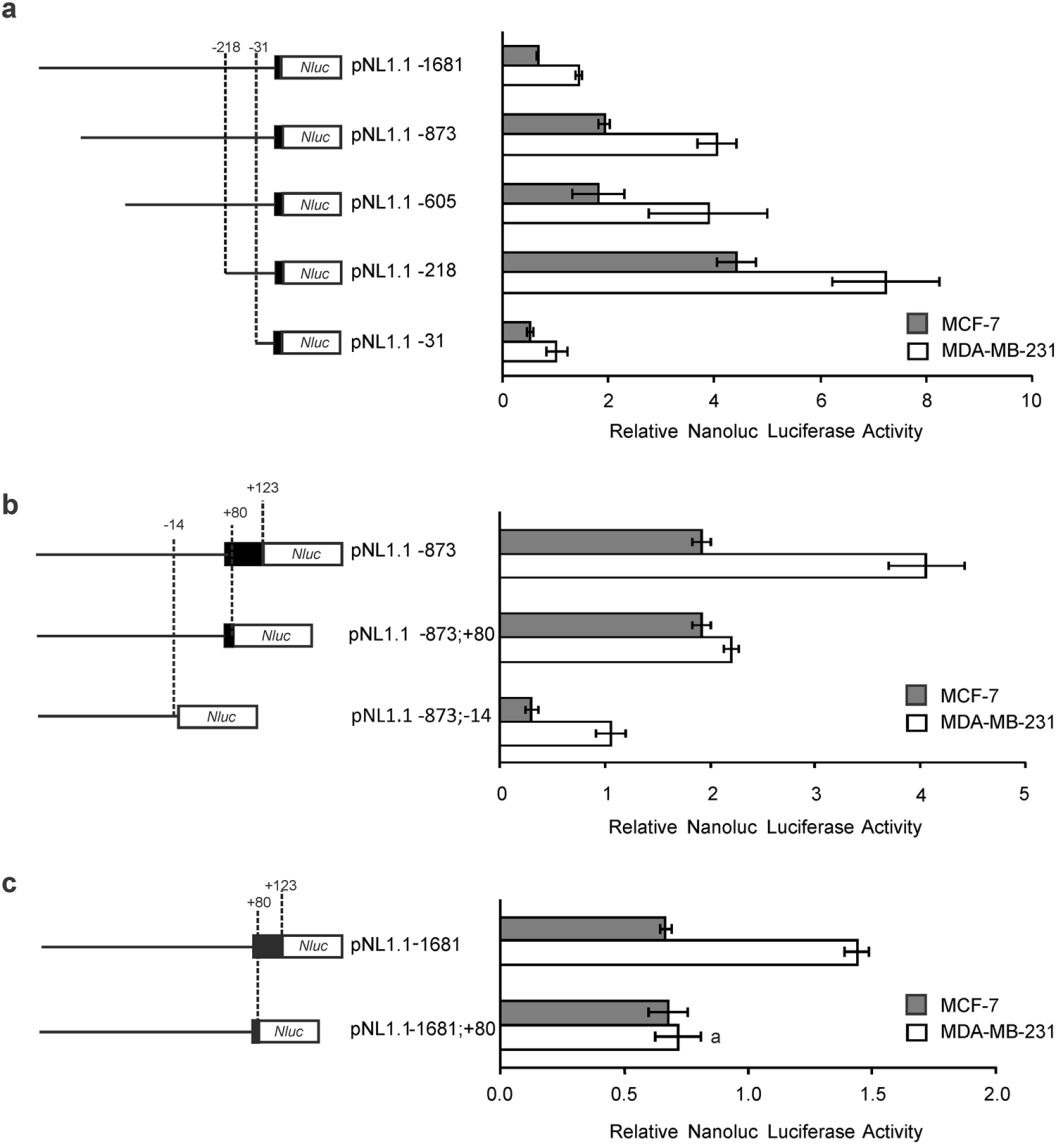
Functional characterization of the *ACSL4* human promoter in MDA-MB-231 and MCF-7 cells. Several constructs of the *ACSL4* promoter cloned upstream of *Nluc* were transfected into MDA-MB-231 and MCF-7 cells. Cells were allowed to recover for 48 h, and Nanoluc Luciferase activity was then determined by luminescence. For comparison between lines, the promoter activity was relativized to the mean of the luminescence units of the empty pNL1.1 vector. (**a**) Results of transfection of a series of plasmids containing 5′ unidirectional deletions of the *ACSL4* promoter (pNL1.1 −1681, −873, −605, −218, −31). Schematic structure of constructs is shown, and positions −31 and −218 of the *ACSL4* promoter are indicated. (**b**) Results of transfection of another series of plasmids containing 3′ unidirectional deletions of a fragment of the *ACSL4* promoter (pNL1.1 −873, −873; +80, −873; −14). Schematic structure of constructs is shown, and positions −14, +80 and +123 of the sequence are indicated. (**c**) Results of transfection of pNL1.1 −1681 or pNL1.1−1681; +80 plasmids. Schematic structure of constructs is shown, and positions +80 and +123 of the sequence are indicated. Results are expressed as the mean +/− SEM arbitrary units of at least three independent experiments. a ***p < 0.001 vs pNL1.1–1681 of MDA-MB-231 cells.

For further studies, we chose two widely used cell lines, MDA-MB-231 and MCF-7 cells, as models of TNBC and ERα-positive breast cancer cells, respectively. To measure the activity of potential *cis*-acting elements, a series of reporter constructs were generated with progressively larger deletions from the 5′ end of the promoter. The activity observed in all constructs was significantly higher in MDA-MB-231 than in MCF-7 cells (Fig. 3a). The results obtained show that the promoter construction carrying a deletion of the 5′ end from position −1681 to −873 (pNL1.1-873) increased promoter activity by 2.8 times in both cell lines, suggesting that this region contains a negative regulatory element (p < 0.001), while the next deletion to position −605 did not generate significant changes. A further deletion to position −218 produced an increase in promoter activity in both lines (p < 0.001), being the most active construction of the series (Fig. 3a). However, in this deletion, the increase in activity was different for each cell line in contrast to what was observed for the other constructions up to here. The increases in promoter activity were 1.8-fold in MDA-MB-231 and a 2.4-fold increase in MCF-7 cells. Therefore, in this case, the difference in promoter activity between lines was smaller, which suggests that between positions −605 and −218 there may be at least one regulatory element of transcription acting differentially between the two cell lines under study. A next deletion to position −31 drastically reduced transcriptional activity (p < 0.001), as the signal fell to values roughly 30% below those observed for the entire sequence (Fig. 3a). These findings suggest that the fragment between −218 and −31 contains elements that positively regulate the basal expression of the gene.

To continue with the functional characterization of the promoter we made progressive deletions of the 3′ end choosing the pNL1.1 −873 plasmid, more active than pNL1.1-1681. Results show that the deletion from position +123 to +80 reduced transcriptional activity by half in MDA-MB-231 (p < 0.001) but not in MCF-7, demonstrating that this sequence fragment is relevant to activate the transcription of the *ACSL4* gene only in MDA-MB-231 (Fig. 3b). A subsequent deletion to −14 position produced a great fall in transcriptional activity in both cell lines respect of pNL1.1 −873 (p < 0.001) (Fig. 3b).

To confirm the effect observed, we generated a plasmid containing the sequence from position −1681 to +80, which was compared to the pNL1.1-1681 construct in both cell lines. The results obtained were in agreement with those observed in deletions on the plasmid pNL1.1 −873. The deletion of 43 bp from the 3′ end also reduced transcriptional activity by 50% only in the MDA-MB-231 cell line (p < 0.001), while transcriptional activity in MCF-7 cells remained unaltered (Fig. 3c). These results confirm that the first 43 bp of the promoter are involved in the increase in ACSL4 expression in MDA-MB-231 cells.

### Sequence analysis of the human *ACSL4* promoter and site-directed mutation effects of selected *cis*-elements

We performed an *in silico* analysis of the human *ACSL4* promoter sequence with MatInspector software (http://www.genomatix.com). The analysis revealed the absence of TATA box and the presence of GC-rich sequences near the transcription initiation site as previously described^11^. With respect to *cis* elements, the analysis revealed several putative transcription factor binding sites (Supplementary Table S1). Considering a cut-off index higher than 85% and bibliographic evidence of transcription factor functionality, we selected Retinoid-related orphan receptor alpha (RORα), Sp1, E2F, and ERRα elements as potential transcriptional regulators of human *ACSL4* promoter. The analysis revealed no consensus sites for ERα.

Site-directed mutagenesis of the selected elements was performed on the pNL1.1-1681 construct.

**Figure 4:**
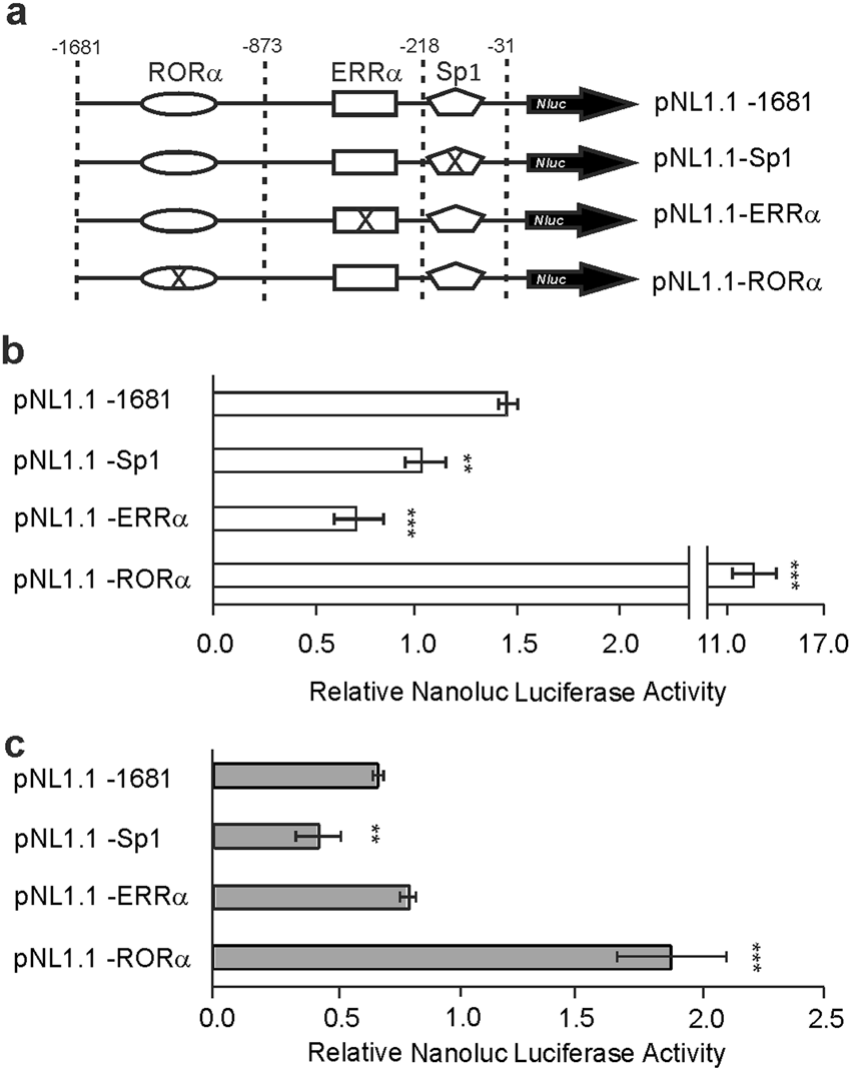
Analysis of Sp1, ERRα and RORα binding sites by site-directed mutagenesis on MDA-MB-231 and MCF-7 cells. Mutants were generated by site-directed mutagenesis of the Sp1 (pNL1.1–Sp1), ERRα (pNL1.1–ERRα), and RORα (pNL1.1–RORα) binding sites present in the pNL1.1 −1681 construct of the human *ACSL4* promoter (**a**) and were transfected into MDA-MB-231 (**b**) and MCF-7 (**c**) cells. Cells were allowed to recover for 48 h, and Nanoluc Luciferase activity was then determined by luminescence. For comparison between lines, the promoter activity was relativized to the mean of the luminescence units of the empty pNL1.1 vector. Results are expressed as the mean +/− SEM arbitrary units of at least three independent experiments. **p < 0.01; ***p < 0.001 *vs* pNL1.1–1681.

RORα is a transcription factor involved in the regulation of lipid metabolism and was suggested as a potential tumor suppressor^12^. A consensus site for RORα was located between positions −1359 to −1326 whose mutation increased promoter activity in both cell lines, suggesting that this factor may act as a repressor (Fig. 4b,c). These results correlate with those observed in the deletion of the 5′ end, region in which the RORα site is present.

Two binding sites were also found on the promoter for transcription factor Sp1, which is involved in the formation of transcription initiation complexes in TATA-less promoters. One of these sites was located between positions −130 and −110 and another between sites −53 and −37. A mutation of the consensus sequence for Sp1 extending between positions −130 and −110 reduced promoter activity in both cell lines, suggesting an activating role of Sp1 (Fig. 4b,c). This result coincides with the activity observed in the deletional analysis in both cell lines for the promoter fragment between −218 and −31 positions, suggesting that Sp1 could be a transcription factor potentially involved in the basal activity of the human *ACSL4* promoter.

**Figure 5:**
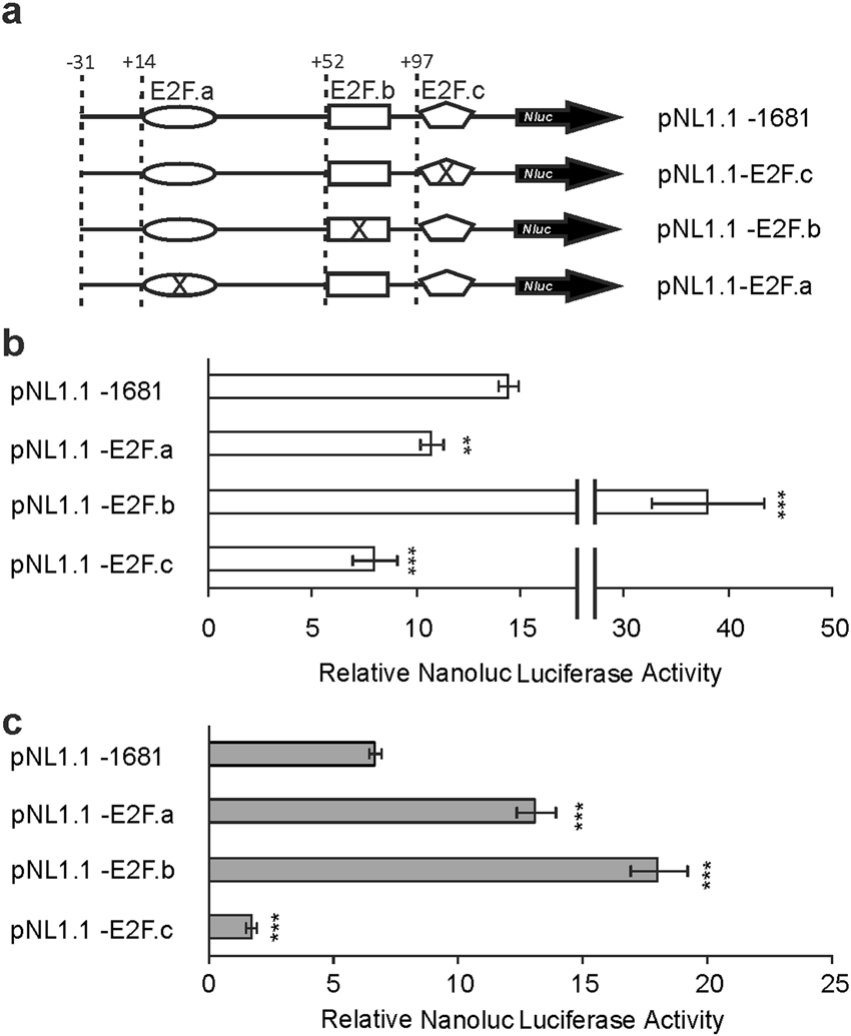
Analysis of E2F binding sites by site-directed mutagenesis on MDA-MB-231 and MCF-7 cells. Mutants were generated by site-directed mutagenesis of the E2F.a (pNL1.1–E2F.a), E2F.b (pNL1.1–E2F.b) and E2F.c (pNL1.1–E2F.c) binding sites present in the pNL1.1 −1681 construct of the human *ACSL4* promoter (**a**) and were transfected into MDA-MB-231 (**b**) and MCF-7 (**c**) cells. Cells were allowed to recover for 48 h, and Nanoluc Luciferase activity was then determined by luminescence. For comparison between lines, the promoter activity was relativized to the mean of the luminescence units of the empty pNL1.1 vector. Results are expressed as the mean +/− SEM arbitrary units of at least three independent experiments. **p < 0.01; ***p < 0.001 *vs* pNL1.1–1681.

The E2F transcription factor family is involved in the regulation of cell cycle progression^13^. Although several E2F consensus sites were found in the entire extension of the promoter, our studies focused on the three consensus sites located near the 3′ end of the promoter between positions +14 to +35, +52 to +69 and +97 to +113, from now referred to as E2F.a, E2F.b and E2F.c, respectively. The mutation for E2F.c decreased promoter activity in both cell lines, thus indicating an activating role in both cell lines under study (Fig. 5b,c). The E2F.b site generated an increase in promoter activity in both cell lines, which hints at a repressive role for this consensus site in the two study models (Fig. 5b,c). The mutant construct E2F.a site may have an activating effect in MDA-MB-231 but a repressive one in MCF-7 cells (Fig. 5b,c). However, these results do not correlate with the activity observed in the 3′ end deletional analysis of the promoter.

**Figure 6:**
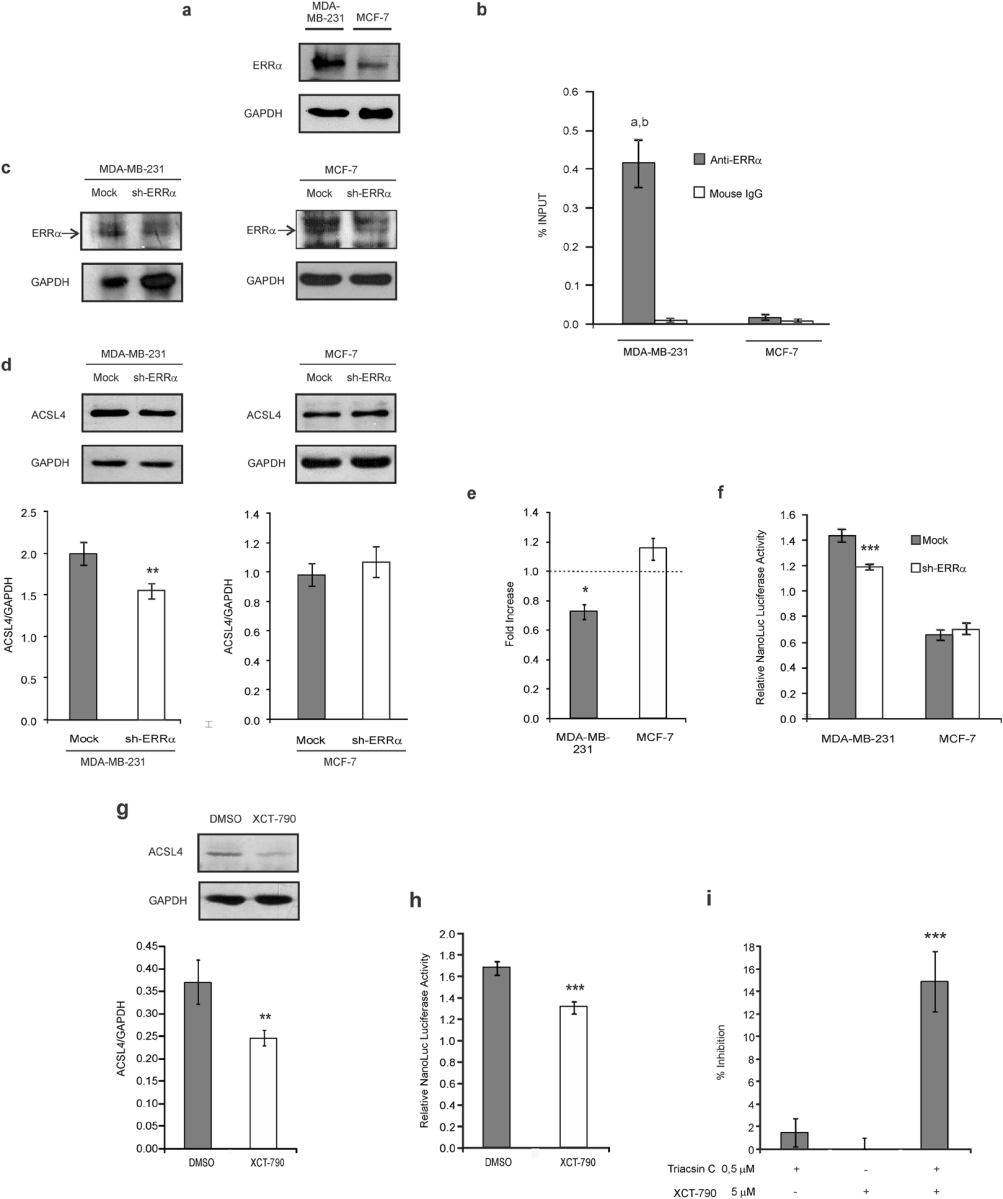
Effect of ERRα on ACSL4 expression in breast cancer cells. (**a**) ERRα expression was analyzed by Western blot. (**b**) ChIP assay was performed from MDA-MB-231 or MCF-7 samples and immunoprecipitated with anti-ERRα antibody or normal mouse IgG as negative control. DNA was recovered and subjected to qPCR analysis using specific primers for the *ACSL4* promoter region. qPCR results are expressed as % of input signal. a ***p < 0.001 *vs* negative controls, b ***p < 0.001 *vs* MCF-7. (**c**) MDA-MB-231 or MCF-7 cells were transiently transfected with the plasmid containing shRNA-ERRα (sh-ERRα) or the empty plasmid (Mock). (**d**) Then ACSL4 was evaluated by Western blot and (**e**) mRNA levels by qPCR. (**f**) pNL1.1–1681 construct was co-transfected either with shRNA-ERRα or empty plasmid (mock) into MDA-MB-231 or MCF-7 cells. Nanoluc luciferase activity was determined by luminescence. Results are expressed as the mean +/− SEM arbitrary units of at least three independent experiments. *p < 0.05, **p < 0.01, ***p < 0.001 *vs* Mock. (**g**) MDA-MB-231 cells were cultured in steroid-free medium and treated with 10 μM XCT-790 or DMSO as vehicle for 24 h. ACSL4 expression was analyzed by Western blot. **p < 0.01 *vs* DMSO. (**h**) MDA-MB-231 cells cultured in steroid-free medium were transfected with the pNL1.1-1681 construct and treated with 10 μM XCT-790 or DMSO for 24 h. The activity of NanoLuc Luciferase was then determined by luminescence. The promoter activity was relativized to the mean of the luminescence units of the empty pNL1.1 vector. Results are expressed as the mean +/− SEM arbitrary units of at least three independent experiments. ***p < 0.001 *vs* DMSO. (**i**) MDA-MB-231 cells were plated at a density of 1200 cells/well in 96-well plates with complete D-MEM and incubated overnight. The medium was then changed to serum-free medium. After 24 h, the cells were switched to 10% FBS-supplemented D-MEM medium with triacsin C (0,5 μM) and/or XCT-790 (5 μM) for 96 h. DMSO was used as vehicle. Subsequently, cell proliferation was measured by BrdU incorporation assays. Results are expressed as the mean +/− SEM arbitrary units of at least three independent experiments. ***p < 0.001 *vs* single inhibitors.

Most interestingly, bioinformatics analysis also showed that the human *ACSL4* promoter sequence contains a site for the ERRα transcription factor. The finding of this consensus site between positions −317 and −293 was of specially interest, as high expression of ERRα is associated with greater aggressiveness and is considered a negative phenotype in the prognosis of breast cancer^14–16^. The expression of ERRα is higher in MDA-MB-231 cells compared to the MCF-7 cell line (Fig. 6a) in line with previous report^15^. The mutation performed on the consensus site for ERRα significantly reduced promoter activity by half in the MDA-MB-231 cell line but exerted no effects in MCF-7 cells (Fig. 4b,c). These results may indicate that ERRα acts as an activator only in the MDA-MB-231 cell line and coincide with the differential increment in signal between lines observed in the deletion from −605 to −218 position in the 5′ deletional analysis.

### Analysis of the role of ERRα on ACSL4 expression

Chromatin inmmunoprecipitation (ChIP) assays were conducted to further analyze the association of ERRα to the *ACSL4* promoter sequence. Cross-linked sheared chromatin from MDA-MB-231 or MCF-7 cells was immunoprecipitated with anti-ERRα or anti-mouse IgG and the DNA recovered was subjected to real-time PCR. A PCR product specific to the *ACSL4* promoter was amplified from anti-ERRα immunoprecipitated DNA samples from MDA-MB-231 cells. In contrast, no significant amplification was observed with DNA recovered from immunoprecipitation using control IgG in this cell line. We observed a very low amplification signal from anti-ERRα immunoprecipitated DNA samples from MCF-7 cells compared to results observed in MDA-MB-231. The signal observed was similar to the obtained from samples using IgG control. These data clearly show the specific association of ERRα to the *ACSL4* promoter in MDA-MB-231 cells.

We next disrupted the expression of endogenous ERRα using small hairpin RNA (shRNA) in order to analyze the effect on ACSL4 expression in MCF-7 and MDA-MB-231 cells. The shRNA targeting ERRα knocked down the expression of the protein (Fig. 6c), which in turn resulted in inhibition of ACSL4 promoter activity (Fig. 6f), mRNA levels (Fig. 6e) and protein expression (Fig. 6d) when compared to mock-transfected cells in MDA-MB-231 cell line. No significant differences were observed in MCF-7 cell line (Fig. 6d–f). These results demonstrate for the first time the role of ERRα as an activator of ACSL4 transcription in MDA-MB-231 cells.

To further explore the role of ERRα in MDA-MB-231 cells we used its well recognized inverse agonist XCT-790^14,15,17^ and analyzed protein expression and promoter activity of the *ACSL4* gene. Incubation with XCT-790 produced inhibition in ACSL4 levels analyzed by Western blot and also in promoter activity (Fig. 6g,h).

On the basis of results obtained regarding the action of ERRα on ACSL4 transcriptional activity and hence its expression, we next evaluated the effect of a combination of ERRα and ACSL4 inhibitors on MDA-MB-231 cell proliferation by combining submaximal concentrations of XCT-790 and triacsin C as ACSL4 inhibitor^18,19^ and subsequently assessing BrdU incorporation. The combination of both drugs generated a significant inhibition in cell proliferation, in contrast to single inhibitor treatment, which rendered no significant effects. These results demonstrate the synergistic inhibitory effect of the joint inhibitors of ERRα and ACSL4 (Fig. 6i).

### Effect of restoring ERα expression in TNBC cells on ACSL4 expression

**Figure 7:**
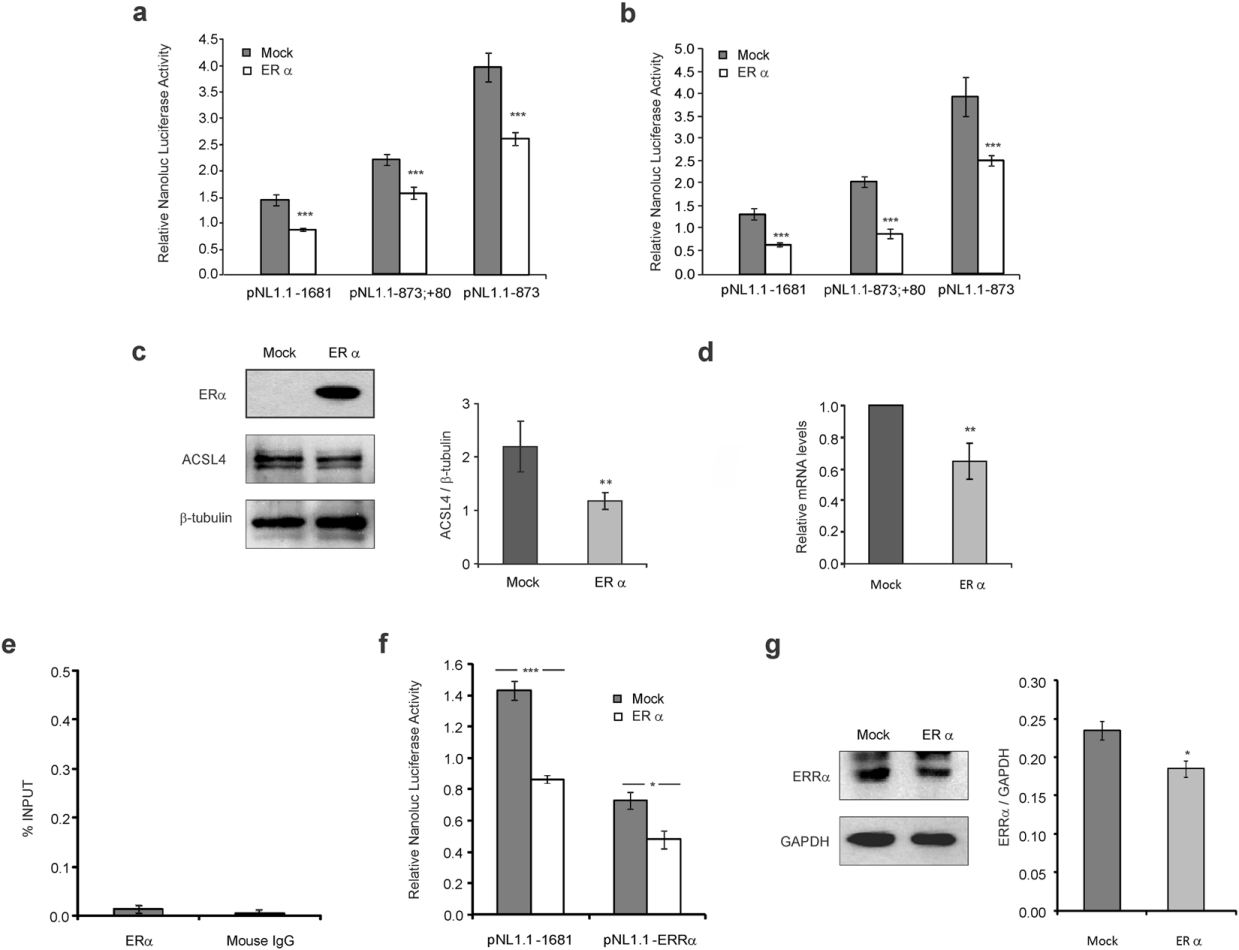
Effect of ERα expression restoration on *ACSL4* expression and promoter activity. Three different constructs of the *ACSL4* promoter (pNL1.1 −1681, pNL1.1 −873; +80 and pNL1.1 −873) were co-transfected either with the pSG5-ERα (ERα) expression plasmid or pSG5 (Mock) empty plasmid into MDA-MB-231 cells (**a**) or Hs578T (**b**) cells. Cells were allowed to recover for 48 h, and Nanoluc luciferase activity was then determined by luminescence. For the analysis of ACSL4 expression and mRNA levels, MDA-MB-231 cells were transiently transfected either with ERα expression plasmid (ERα) or empty vector (Mock) and allowed to recover for 48 h. (**c**) ERα and ACSL4 expression was then analyzed by Western blot. ACSL4 signal was quantified by densitometry. Integrated optical density of ACSL4 was normalized against β-tubulin expression. (**d**) Total RNA was extracted, reverse-transcribed and ACSL4 and L19 human ribosomal protein (L19) mRNAs were subjected to real-time PCR. ACSL4 mRNA levels were normalized against the corresponding L19 mRNA levels. Results are expressed as the mean +/− SEM arbitrary units of at least three independent experiments. **p < 0.01*vs* Mock. (**e**) ChIP assay was performed using cross-linked sheared chromatin obtained from MCF-7 and immunoprecipitated with anti-ERα antibody. Immunoprecipitation with normal mouse IgG was run as negative control. DNA was recovered and subjected to qPCR analysis using specific primers for the *ACSL4* promoter region. qPCR results are expressed as % of input signal. (**f**) The constructs pNL1.1–1681or pNL1.1–ERRα were co-transfected either with the pSG5-ERα (ERα) expression plasmid or pSG5 (Mock) empty plasmid into MDA-MB-231 cells. Cells were allowed to recover for 48 h, and Nanoluc luciferase activity was then determined by luminescence. Results are expressed as the mean +/− SEM arbitrary units of at least three independent experiments. *p < 0.05,***p < 0.001 *vs* Mock. (**g**) ERRα expression was analyzed by Western blot in MDA-MB-231 transfected with the pSG5-ERα (ERα) expression plasmid or pSG5 (Mock) empty plasmid. Integrated optical density of ERRα was normalized against GAPDH expression. Results are expressed as the mean +/− SEM arbitrary units of at least three independent experiments. *p < 0.05 *vs* Mock.

Previously, we have reported an inverse relationship between ACSL4 and ERα expression both *in vitro* and *in vivo*^3^. On the other hand, ERα silencing has been shown to enhance ACSL4 expression in MCF-7^5^. However, our bioinformatics analysis of the *cis*-elements of the human *ACSL4* promoter did not show consensus sequences for ERα. Therefore, to demonstrate that ERα regulates ACSL4 expression at transcriptional level, we restored the expression of this transcription factor in TNBC cells to analyze its effect on *ACSL4* promoter activity, transcription and expression. We cotransfected the plasmid pSG5-ERα^20^ or its corresponding empty plasmid pSG5 into TNBC cell lines MDA-MB-231 (Fig. 7a) and Hs578T (Fig. 7b), with different constructions of *ACSL4* promoter. Results showed ERα expression to reduce the activity of all constructions of the *ACSL4* promoter tested (Fig. 7a,b). In turn, overexpression of ERα in MDA-MB-231 decreased both the expression (Fig. 7c) and mRNA level of ACSL4 (Fig. 7d), as revealed by Western blot and qPCR assays, in agreement with the results obtained in promoter activity.

ChIP assays were conducted on ERα positive MCF-7 cell line to further analyze the association of ERα to the *ACSL4* promoter sequence (Fig. 7e). Cross-linked sheared chromatin from MCF-7 cells was immunoprecipitated with anti-ERα or anti-mouse IgG and the DNA recovered was subjected to real-time PCR. We observed a very low amplification signal from anti-ERα immunoprecipitated DNA samples. The signal observed was similar to the obtained from samples using IgG control. These data do not support the association of ERα to the *ACSL4* promoter sequence (Fig. 7e). Following the analysis, we studied the effect of restoring ERα on ERRα protein levels in MDA-MB-231 cells. We observed that the expression of ERα produces a decrease in the expression levels of ERRα (Fig. 7g). Then we analyze the effect of ERα on the promoter activity containing the site directed mutation for ERRα. We observed that ERα expression produces a decrease in the activity of the construction that contains a mutation of the ERRα site, though slighter than the decrease observed in the wild type sequence (Fig. 7f).

## Discussion

Here we demonstrate for the first time that differential transcriptional activity is involved in the regulation of ACSL4 expression in breast cancer cell lines. We cloned and functionally characterized the human *ACSL4* promoter in breast cancer cell lines and was functional in all the lines studied, exhibiting greater activity in TNBC cells. Both differences and common features were found in *ACSL4* promoter regulation of the systems under study. Within the similarities observed, deletional analysis revealed that the sequence between −1681 and −873 positions contains at least one negative regulator. Within this area a consensus site was found for RORα, which in normal cells is involved in the regulation of functions such as lipid metabolism^21^. Several actions for RORα have also been found in melanoma and breast cancer^22,23^, and the presence of RORα mRNA has been reported both in TNBC and ER-positive human breast cancer cells^24^. Through site-directed mutagenesis, we show that RORα may act as a negative regulator of the *ACSL4* promoter. Given that lower expression of ACSL4 has been associated with lower aggressiveness in breast cancer^1,3^, the negative regulation of the *ACSL4* promoter by RORα observed here is in line with the potential role of this transcription factor as a tumor suppressor^12^.

Adding up to the similarities observed, the sequence between −218 and −31 was found to be a positive regulatory zone. This result confirms that this fragment is responsible for most of the activity of the human promoter and might contain the necessary elements for basal transcription. The first 230 bp of the *ACSL4* promoter are highly conserved (85% homology) between human and mouse species^11^, and consensus sites for Sp1 have been described in this region^11,25^. However, until now, no experimental data were available on the functionality of these elements in the human *ACSL4* promoter. Sp1 is of particular interest, as it has been described as a CG binding transcription activator participating in the formation of transcription initiation complexes^26^. Site-directed mutagenesis of the Sp1 consensus sequence located between −130 and −110 positions indicated that it functions as an activator.

Most interestingly, we discovered differences in promoter functionality which were involved in the increase in ACSL4 transcriptional activity in MDA-MB-231 cells. In the current work, the 3′ deletional analysis demonstrated that the 43 bp of the 3′ end of the *ACSL4* promoter are relevant to the higher promoter activity, as the deletion of these bases surprisingly generated a fall which drove it to the same levels of MCF-7 cells. E2F.c consensus site was located in this region. However, the analysis of E2F.c element indicates that it acts as an activator in both lines, which suggests that may not itself be involved in the differential activity observed in the 3′ end of the promoter. As possible explanations for the differential activity of the proximal 43 bp of the promoter, the deleted fragment may have eliminated alternative transcription starting sites, or different epigenetic modifications on the promoter of each cell line might influence the transcriptional activity; in both cases, the fall in the deletional analysis may not only be associated with the activity of consensus sites themselves.

Also as part of the 3′ deletional analysis, a deletion between −14 and +80 positions of the promoter was found to cause a greater fall in activity in both lines under study. Two additional consensus sequences for the E2F family were found in this region. Site-directed mutations of these *cis*-elements indicate that E2F.b may act as a repressor in both cell lines, while E2F.a might act as an activator in MDA-MB-231 and as a repressor in MCF-7. This difference found could be due to the fact that, in general, several members of the E2F family join a region of a promoter and that binding specificity of a particular factor may depend on the amount present of each member of the family in the nucleus^27^ or on the specific activity of a E2F family member in each cellular context^28^. However, as these results fail to completely support the difference in activity fall observed upon deletion of this fragment, the net effect of E2F may be thought to depend on the balance and interaction between activators and repressors of the E2F family in each cell type.

Most interestingly, results obtained in the 5′ deletional analysis suggest a difference between lines within the −605 and −218 positions. This difference may be explained by an activator probably acting only in MDA-MB-231 cells in this zone. This fragment contains a consensus sequence for ERRα, a transcription factor identified on the basis of its homology with ERα. To exert its action, ERRα requires specific interaction with coactivators or corepressors^29,30^. It has been reported that ERRα is differentially expressed in cancerous cells of different aggressiveness^15^. In correspondence with the expression of ACSL4 in breast cancer^2,3,9^, a negative correlation has been established between expression levels of ERα and ERRα in breast cancer tumor samples^31^. ERRα is considered a negative prognostic marker for the disease, as its expression in breast carcinoma has been associated with increased risk of recurrence and adverse clinical progression^32^. Moreover, ERRα is known to induce the expression of vascular endothelial growth factor in different breast cancer cell lines^33^, and to increase metastatic capacity in breast tumors^15,34^. It has been shown *in vitro* and *in vivo* that ERRα is involved in the epithelial-mesenchymal transition^15^. Like the ACSL4 functions reported in relation to aggressiveness in breast cancer cell lines^1,3,5^, ERRα expression promotes cellular migration, invasion and tumor growth^15^. All this evidence justified our study of ERRα *cis*-element in the *ACSL4* promoter. Site-directed mutagenesis results indicated that ERRα acts as an activator only in MDA-MB-231 cells, which correlates with the deletional analysis of the zone. ERRα binding to the *ACSL4* promoter was confirmed through ChIP assays in MDA-MB-231 cells. In line with these results, inhibition of ERRα expression by shRNA resulted in decreased ACSL4 promoter activity, mRNA and protein levels only en MDA-MB-231 cells. This result demonstrates that ERRα regulates ACSL4 at transcriptional level. Furthermore, we evaluated ERRα role in the regulation of ACSL4 using the specific ERRα inverse agonist XCT-790 and observed that both the activity of the promoter and the expression of ACSL4 decrease significantly upon ERRα inhibition in MDA-MB-231 cells. Altogether, these findings demonstrate for the first time that ERRα is a transcription factor involved in the upregulation of the expression of ACSL4 in MDA-MB-231 cells. In addition, the current work shows that inhibitors of ACSL4 and ERRα reduces the proliferation of MDA-MB-231 cells in a synergistic way. A previous work showed an uncoupling effect of XCT-790 independent of ERRα^35^. We cannot rule out that this effect also could be participating in the observed results.

Additionally, we observed a relationship between ERRα activity and the 43 bp function of the 3′ end of the promoter. Both pNL1.1 −1681; +80 and pNL1.1 −873; +80 contain the consensus site for ERRα; however, the activity of these constructs lacking the 43 bp of the 3′ end did not show significant differences between cell lines, which indicates that ERRα stimulatory effect on the *ACSL4* promoter in MDA-MB-231 may not be maintained when the 43 bp of the 3′ end are deleted. At the same time, it is worth pointing out the need of ERRα binding to maintain the difference between cell lines, given that the mutant construction for ERRα on the whole promoter caused a fall in transcriptional activity in MDA-MB-231 to levels similar to those of MCF-7 cells. This evidence suggests that both the 43 bp of the 3′ end of the promoter and ERRα action might be necessary to produce differential regulation between cell lines of different aggressiveness.

We also evaluated the role of ERα in the regulation of ACSL4. Previous reports have established an inverse relationship between the expression of ACSL4 and ERα in breast cancer, as shown both in cancer cell lines and patients’ breast tumors, and indicate that ACSL4 negatively modulates the expression of ERα^3,5,9^. Furthermore, the silencing of ACSL4 in MDA-MB-231 cells restores ERα expression^2^, which is in line with results obtained by other authors through the overexpression of ACSL4 in MCF-7 cells^5^. Moreover, the regulation of ERα by ACSL4 seems to be reciprocal, as ERα silencing in MCF-7 cells induces ACSL4 expression, which indicates that ERα in turn modulates the expression of ACSL4^5^. However, the effects of ERα restoration on TNBC cells had not been evaluated before. Our current results upon restoring ERα expression in TNBC cells show a decrease in ACSL4 promoter activity. Furthermore, in MDA-MB-231 cells, we demonstrate that the effects observed on the promoter are consequently reflected in a decrease in ACSL4 protein and mRNA levels. In this way, we confirm that this transcription factor negatively regulates ACSL4 expression. As bioinformatic analysis of *cis*-elements on the human ACSL4 promoter revealed no consensus sequences for ERα. In our ChIP experiments we could not detect a significant binding of ERα to the ACSL4 promoter. We hypothesize that this transcription factor may be thought to regulate the ACSL4 promoter by an indirect regulatory mechanism. We observed that the restoration of ERα expression produces a decrease in ERRα protein in MDA-MB-231 cells. We showed that the effect of ERα on the promoter activity persists even when the consensus site of ERRα is mutated, though slighter than the decrease observed in the wild type sequence. We conclude that binding to the promoter sequence may not play a key role in the effect of ERα on the expression of ACSL4, and this effect could be produced in part by the regulation it exerts on ERRα.

In conclusion, the results presented in this manuscript demonstrate that the different expression of ACSL4 in human breast cancer cell lines of different aggressiveness is at least partly due to a different transcriptional regulation. We have established for the first time that ERRα is a transcription factor involved in the higher activation of the human *ACSL4* promoter in TNBC cell lines. Previous studies have proposed ERRα as a possible therapeutic target in breast cancer and its inhibition is currently under study as a new strategy for TNBC treatment^14,15^. Therefore, the future joint study of ACSL4 and ERRα could lead to the design of new drug combinations for greater effectiveness in the treatment of highly aggressive breast cancer. Moreover, we show that ERα exerts a negative regulation on the expression of ACSL4 which might partially explain its overexpression in TNBC. Additional elements on the *ACSL4* promoter with effects on gene transcription remain to be identified in further studies which will help to elucidate the molecular mechanisms underlying abnormal ACSL4 expression in pathological conditions and potentially identify new pathways of regulation.

## Methods

### Materials

Dulbecco’s modified eagle medium (DMEM), Opti-MEM, Lipofectamine 2000, penicillin-streptomycin solution, trypsin-EDTA and PCR primers were purchased from ThermoFisher Scientific (Waltham, MA, USA). Fetal bovine serum (FBS) was from PAA laboratories GmbH (Pasching, Austria). XCT-790 was from Sigma Chemical Co. (St. Louis, MO, USA). Triacsin C was from Enzo Life Sciences (Farmingdale, NY, USA). Polyclonal rabbit antibody anti-ACSL4 was generated in our laboratory^36^. Monoclonal mouse anti-GAPDH and polyclonal rabbit anti-ERα antibodies were from Santa Cruz Biotechnology, Inc. (Dallas, TX, USA). Monoclonal mouse anti-β-tubulin was from Upstate Group Inc (Temecula, CA, USA). Polyclonal rabbit anti-ERRα chromatin immunoprecipitation (ChIP) grade was from Abcam (Cambridge, UK). Horseradish peroxidase-conjugated goat anti-rabbit and goat anti-mouse secondary antibodies and Immuno-Blot PDVF membrane were from Bio-Rad Laboratories (Hercules, CA, USA). Enhanced chemiluminescence (ECL) was from GE Healthcare (Buckinghamshire, UK). Sterile and plastic material for tissue culture was from Orange Scientific (Braine-l′Alleud, Belgium). All other reagents were of the highest grade available.

### Cell culture and treatments

Human breast cancer cell lines MDA-MB-231, Hs578-T, MCF-7 and T47D were generously provided by Dr. Vasilios Papadopoulos (School of Pharmacy, University of Southern California, Los Angeles, CA, USA) or obtained from the Lombardi Comprehensive Cancer Center (Georgetown University Medical Center, Washington D.C., USA). All cell lines were cultured in DMEM with glucose (4500 mg/l) supplemented with heat-inactivated FBS (10%) plus penicillin (100 U/ml) and streptomycin (10 μg/ml) and maintained in a 5% CO_2_ humidified atmosphere. The experiments for ERRα inhibition were performed in steroid free condition as indicated in the literature^14,15^.

### Promoter cloning and Nanoluc luciferase reporter construct generation

Genomic DNA was amplified with the specific primers (Table 1) containing XhoI and HindIII restriction sites. A ~1.8 kb sequence containing the *ACSL4* human promoter was cloned into a pNL1.1 vector (Promega Corp., Madison, WI, USA) creating the plasmid pNL1.1-1681. An equal procedure was followed to obtain plasmids pNL1.1-873, −605, −218 and −31, containing 5′ unidirectional deletions of the promoter or pNL1.1 −873; +80 and pNL1.1 −873;−14 containing 3′ unidirectional deletions of the pNL1.1-873 construct, using the corresponding specific pairs of primers (Table 1). In the same way, the pNL1.1 −1681; +80 plasmid was obtained using the specific primers (Table 1). Reporter constructs pNL1.1–E2F.a, -E2F.b, -E2F.c, –RORα, –Sp1 and –ERRα consist of pNL1.1-1681 carrying mutations in the corresponding putative transcription factor binding sites and were generated with Quik Change Site-Directed Mutagenesis Kit (Agilent Technologies, Santa Clara, CA, USA) following the suppliers’ instructions. All plasmids were fully sequenced for verification purposes (Macrogen, Korea).

**Table 1:** Primers used in the expression and functional analysis of the *ACSL4* promoter.

Reaction	Primer	Primer sequence (5′-3′)
Promoter cloning	ACSL4 promoter forward	AAA CTC GAG TGG GCT AAG AGA CCG ACA ATA
	ACSL4 promoter reverse	TTT AAG CTT GGC ACT CGG AAA GCT CGC AAA
5′ deletions	del −31	AAA CTC GAG GCG TCT TTT CCG GGC TCG
	del −218	AAA CTC GAG GTT AGC CGC CTC GCC TTC
	del −605	AAA CTC GAG ACG TGA AAA TCC TGC GTC CTT
	del −873	AAA CTC GAG GAA TTC TTG AGA TTT ATT TTT TTG G
3′ deletions	del + 80	TTT AAG CTT CGC TGG GAC GAG GAG GA
	del −14	TTT AAG CTT GAG CCC GGA AAA GAC GC
Site-directed mutagenesis	E2F.a forward	GTC CGG GTG CGC AAG CGC CGC TGC GGC
	E2F.a reverse	GCC GCA GCG GCG CTT GCG CAC CCG GAC
	E2F.b forward	GGC CTC CGC CGC GAA CTC CTC CTC GTC C
	E2F.b reverse	GGA CGA GGA GGA GTT CGC GGC GGA GGC C
	E2F.c forward	GCA CGC GGT TCC TTC TTG CGA GCT TTC CG
	E2F.c reverse	CGG AAA GCT CGC AAG AAG GAA CCG CGT GC
	Sp1 forward	CGG GCG AGC GGG TGC GGG CGC GTG G
	Sp1 reverse	CCA CGC GCC CGC ACC CGC TCG CCC G
	ERRα forward	CTG CTG TTA GGC GCA AGG CTA TCC CGA AAC ACA CTC AAC
	ERRα reverse	GTT GAG TGT GTT TCG GGA TAG CCT TGC GCC TAA CAG CAG
	RORα forward	GAA GTA CTG AAC TTT GTT GGT CAA TCT TGA AAA AG
	RORα reverse	CTT TTT CAA GAT TGA CCA ACA AAG TTC AGT ACT TC
qPCR	ACSL4 forward	CCC GCT ATC TCC TCA GAC AC
	ACSL4 reverse	CAA TTT CAT GGC ATT TGC AG
	L19 forward	AGT ATG CTC AGG CTT CAG AA
	L19 reverse	TTC CTT GGT CTT AGA CCT GC
ChIP	ERRα ChIP forward	GTG GAG TCC TGC GAA GCA AG
	ERRα ChIP reverse	CGG CTC CGC CTC AAG TTG TTG

For luciferase reporter assays, cells were plated in 48-well plates (100,000 cells/well). Transfection was performed 24 h later with the different reporter plasmids, using Lipofectamine 2000. All reporter plasmids were used in equimolar amounts, and pBluescript DNA was used as needed to keep the total amount of DNA constant. Plasmid pRc/CMVi (Life Technologies, Carlsbad, CA, USA) expressing the *EGFP* gene was used for normalization^37^. After transfection, total lysates were recovered using passive lysis buffer and luciferase activity was analyzed through the Nano-Glo® Luciferase Assay System (Promega), and luminescense was measured using an automated plate reader (Synergy HT, BioTek, Winooski, VT, USA). Nanoluc luciferase activity was normalized to EGFP fluorescence counts measured in the same equipment and expressed as arbitrary units.

The plasmid sh-ERRα was generated by cloning the annealed primers 5′GATCCCCCTCGGAGACAGAGACCGAGTTCAAGAGACTCGGTCTCTGTCTCCGAGTTTTTA 3′ and 5′ AGCTTAAAAACTCGGAGACAGAGACCGAGTCTCTTGAACTCGGTCTCTGTCTCCGAGGGG 3′ into pSUPER.retro.puro as previously described^1^. The plasmid PSG5-ERα^20^ was used for restoring ERα expression.

### Chromatin immunoprecipitation (ChIP) assay

ChIP assays were performed following the manufacturer’s protocol of the EZ-ChIP assay kit (EMD Millipore Corporation, Billerica, MA, USA) as previously described^11^. Briefly, formaldehyde was added to cell cultures to a final concentration of 1% for 10 min at 37 °C. After the addition of glycine, cells were collected, pelleted and resuspended in the provided SDS-lysis buffer (6 × 10^6^ cells/100 µl). For chromatine fragmentation, sonication was performed using a Branson Sonifier 250 (VWR International, Radnor, PA, USA) for 6 cycles of 10 s pulses with an amplitude of 10%. Chromatin fragments were determined to be between 200–1000 bp in size. Sheared chromatin was immunoprecipitated overnight with anti-ERRα antibody. Immunoprecipitation with normal mouse IgG or absence of antibody were run as negative controls. Immunocomplexes were collected using salmon sperm/protein A-agarose slurry and subsequently washed and eluted using SDS containing elution buffer. Formaldehyde cross-links of samples, controls and input were reversed and DNA was purified. Real time PCR was performed using promoter-specific primers (Table 1). The bound DNA was calculated in equation 1 as % input using the Ct values as previously described^38^. The formula used was the following:

1 $$\begin{array}{rcl}{\rm{\Delta }}\mathrm{Ct}[{\rm{adjusted}}\,{\rm{ChIP}}] & = & {\rm{Ct}}[{\rm{ChIP}}]-({\rm{Ct}}[{\rm{Input}}]\\  &  & -{\mathrm{Log}}_{2}({\rm{Input}}\,{\rm{Dilution}}\,{\rm{Factor}})).\\  \% \,{\rm{Input}} & = & 100/{2}^{{\rm{\Delta }}\mathrm{Ct}[{\rm{adjusted}}{\rm{ChIP}}]}\end{array}$$

### RNA extraction and real-time PCR

For quantitative real-time PCR total RNA was extracted using Tri Reagent following the manufacturer’s instructions (Molecular Research Center Inc., Cincinnati, OH, USA). Isolated RNA was deoxyribonuclease-treated using RNAse-free DNase RQ1 (Promega). Reverse transcription was done using total RNA (2 μg) and M-MLV Reverse Transcriptase (Promega). Reactions were carried out using the SYBR Green Master Mix reagent kit (Applied Biosystems, Carlsbad, CA, USA) and using the specific primers for qPCR (Table 1). The reaction conditions were: one 5 min cycle at 95 °C, followed by 40 cycles at 95 °C for 15 s, 60 °C for 30 s, and 72 °C for 30 s. Gene mRNA expression levels were normalized to human L19 RNA expression, performed in parallel as endogenous control. Real-time PCR data were analyzed by calculating the 2−ΔΔCt value (comparative Ct method) for each experimental sample.

### Western blot

Western blot was performed as previously described^36^. Appropriate dilutions of primary antibodies were used as recommended by the manufacturer. The polyclonal rabbit antibody anti-ACSL4 generated in our laboratory was used in a dilution of 1:1000^36^.

### Cell proliferation assays

Cell proliferation was measured by 5-bromo-2′-deoxyuridine (BrdU) incorporation using BrdU cell proliferation ELISA kit (Roche Diagnostics, Basel, Switzerland) following the manufacturer’s instructions as previously described^2^.

### Sequence and statistical analysis

Sequence analysis was performed with Vector NTi (Informax, Invitrogen, Carlsbad, CA, USA). Identification of putative transcription factor binding sites was achieved using MatInspector version 2.2 (Genomatix, Inc., Munich, Germany). Statistical analyses were performed with PRISM version 3.0 (GraphPad, Inc., La Jolla, CA, USA).

## Supplementary information

Dataset 1

Suppelementary Information

## References

[1] Maloberti PM (2010). Functional interaction between acyl-CoA synthetase 4, lipooxygenases and cyclooxygenase-2 in the aggressive phenotype of breast cancer cells. PloS one.

[2] Orlando UD (2015). Acyl-CoA synthetase-4, a new regulator of mTOR and a potential therapeutic target for enhanced estrogen receptor function in receptor-positive and -negative breast cancer. Oncotarget.

[3] Orlando UD (2012). The functional interaction between Acyl-CoA synthetase 4, 5-lipooxygenase and cyclooxygenase-2 controls tumor growth: a novel therapeutic target. PloS one.

[4] Wu X (2015). ACSL4 promotes prostate cancer growth, invasion and hormonal resistance. Oncotarget.

[5] Wu X (2013). Long chain fatty Acyl-CoA synthetase 4 is a biomarker for and mediator of hormone resistance in human breast cancer. PloS one.

[6] Xia H (2017). Simultaneous silencing of ACSL4 and induction of GADD45B in hepatocellular carcinoma cells amplifies the synergistic therapeutic effect of aspirin and sorafenib. Cell death discovery.

[7] Cao Y, Dave KB, Doan TP, Prescott SM (2001). Fatty acid CoA ligase 4 is up-regulated in colon adenocarcinoma. Cancer research.

[8] Sun XJ, Xu GL (2017). Overexpression of Acyl-CoA Ligase 4 (ACSL4) in Patients with Hepatocellular Carcinoma and its Prognosis. Medical science monitor: international medical journal of experimental and clinical research.

[9] Monaco ME (2010). Expression of Long-chain Fatty Acyl-CoA Synthetase 4 in Breast and Prostate Cancers Is Associated with Sex Steroid Hormone Receptor Negativity. Translational oncology.

[10] Orlando UD (2018). Acyl-CoA synthetase-4 is implicated in drug resistance in breast cancer cell lines involving the regulation of energy-dependent transporter expression. Biochemical pharmacology.

[11] Orlando U (2013). Characterization of the mouse promoter region of the acyl-CoA synthetase 4 gene: role of Sp1 and CREB. Molecular and cellular endocrinology.

[12] Du J, Xu R (2012). RORalpha, a potential tumor suppressor and therapeutic target of breast cancer. International journal of molecular sciences.

[13] Haase SB, Wittenberg C (2014). Topology and control of the cell-cycle-regulated transcriptional circuitry. Genetics.

[14] Wu YM (2016). Inverse agonist of estrogen-related receptor alpha suppresses the growth of triple negative breast cancer cells through ROS generation and interaction with multiple cell signaling pathways. Oncotarget.

[15] Wu YM (2015). Inhibition of ERRalpha suppresses epithelial mesenchymal transition of triple negative breast cancer cells by directly targeting fibronectin. Oncotarget.

[16] Casaburi I (2018). Cholesterol as an Endogenous ERRalpha Agonist: A New Perspective to Cancer Treatment. Frontiers in endocrinology.

[17] Du Y (2017). The discovery of novel, potent ERR-alpha inverse agonists for the treatment of triple negative breast cancer. European journal of medicinal chemistry.

[18] Tomoda H, Igarashi K, Omura S (1987). Inhibition of acyl-CoA synthetase by triacsins. Biochimica et biophysica acta.

[19] Cornejo Maciel F (2005). An arachidonic acid-preferring acyl-CoA synthetase is a hormone-dependent and obligatory protein in the signal transduction pathway of steroidogenic hormones. Journal of molecular endocrinology.

[20] Tora L (1989). The cloned human oestrogen receptor contains a mutation which alters its hormone binding properties. The EMBO journal.

[21] Kim K (2017). RORalpha controls hepatic lipid homeostasis via negative regulation of PPARgamma transcriptional network. Nature communications.

[22] Brozyna AA, Jozwicki W, Skobowiat C, Jetten A, Slominski AT (2016). RORalpha and RORgamma expression inversely correlates with human melanoma progression. Oncotarget.

[23] Xiong G, Wang C, Evers BM, Zhou BP, Xu R (2012). RORalpha suppresses breast tumor invasion by inducing SEMA3F expression. Cancer research.

[24] Dai J, Ram PT, Yuan L, Spriggs LL, Hill SM (2001). Transcriptional repression of RORalpha activity in human breast cancer cells by melatonin. Molecular and cellular endocrinology.

[25] Minekura H (2001). Exon/intron organization and transcription units of the human acyl-CoA synthetase 4 gene. Biochemical and biophysical research communications.

[26] Emami KH, Burke TW, Smale ST (1998). Sp1 activation of a TATA-less promoter requires a species-specific interaction involving transcription factor IID. Nucleic acids research.

[27] Xu X (2007). A comprehensive ChIP-chip analysis of E2F1, E2F4, and E2F6 in normal and tumor cells reveals interchangeable roles of E2F family members. Genome research.

[28] Chong JL (2009). E2f1-3 switch from activators in progenitor cells to repressors in differentiating cells. Nature.

[29] Brown EL (2014). PGC-1alpha and PGC-1beta increase CrT expression and creatine uptake in myotubes via ERRalpha. Biochimica et biophysica acta.

[30] You J (2017). Receptor-interacting Protein 140 represses Sirtuin 3 to facilitate hypertrophy, mitochondrial dysfunction and energy metabolic dysfunction in cardiomyocytes. Acta physiologica.

[31] Ariazi EA, Clark GM, Mertz JE (2002). Estrogen-related receptor alpha and estrogen-related receptor gamma associate with unfavorable and favorable biomarkers, respectively, in human breast cancer. Cancer research.

[32] Suzuki T (2004). Estrogen-related receptor alpha in human breast carcinoma as a potent prognostic factor. Cancer research.

[33] Stein RA, Gaillard S, McDonnell DP (2009). Estrogen-related receptor alpha induces the expression of vascular endothelial growth factor in breast cancer cells. The Journal of steroid biochemistry and molecular biology.

[34] Fradet A (2011). Dual function of ERRalpha in breast cancer and bone metastasis formation: implication of VEGF and osteoprotegerin. Cancer research.

[35] Eskiocak B, Ali A, White MA (2014). The estrogen-related receptor alpha inverse agonist XCT 790 is a nanomolar mitochondrial uncoupler. Biochemistry.

[36] Castillo F (2004). Tyrosine phosphates act on steroidogenesis through the activation of arachidonic acid release. Endocrine research.

[37] Vesuna F, Winnard P, Raman V (2005). Enhanced green fluorescent protein as an alternative control reporter to Renilla luciferase. Analytical biochemistry.

[38] Lin X, Tirichine L, Bowler C (2012). Protocol: Chromatin immunoprecipitation (ChIP) methodology to investigate histone modifications in two model diatom species. Plant methods.

