# Supplementary material for: Regulatory mechanisms leading to differential Acyl-CoA synthetase 4 expression in breast cancer cells
Source: Sci Rep. 2019 Jul 16;9:10324. doi: 10.1038/s41598-019-46776-7 (PMC6635356; doi:10.1038/s41598-019-46776-7)
Supplement: Supplementary file 2 — Suppelementary Information [file 41598_2019_46776_MOESM2_ESM.pdf]

Supplementary Information Guide

To accompany submitted manuscript entitled: **"Regulatory mechanisms leading to differential Acyl-CoA synthetase 4 expression in breast cancer cells"** (Melina A. Dattilo, Yanina Benzo, Lucía M. Herrera, Jesica G. Prada, Ana F. Castillo, Ulises D. Orlando, Ernesto J. Podesta and Paula M. Maloberti)

Supplementary Figure S1. Full-length blots

Supplementary Figure S1. Full-length blots

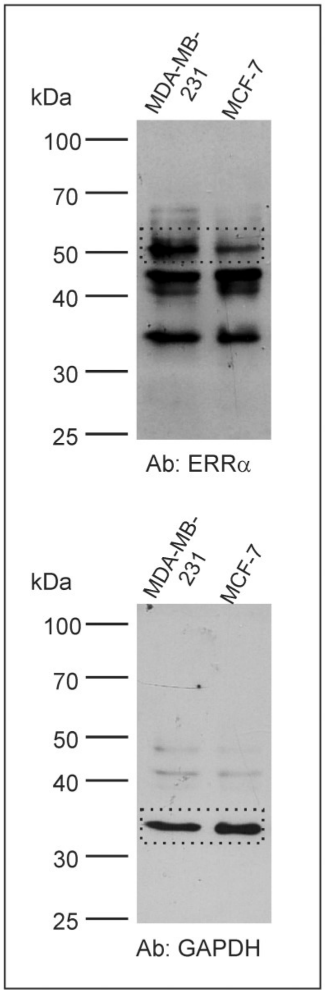

Figure 6 a

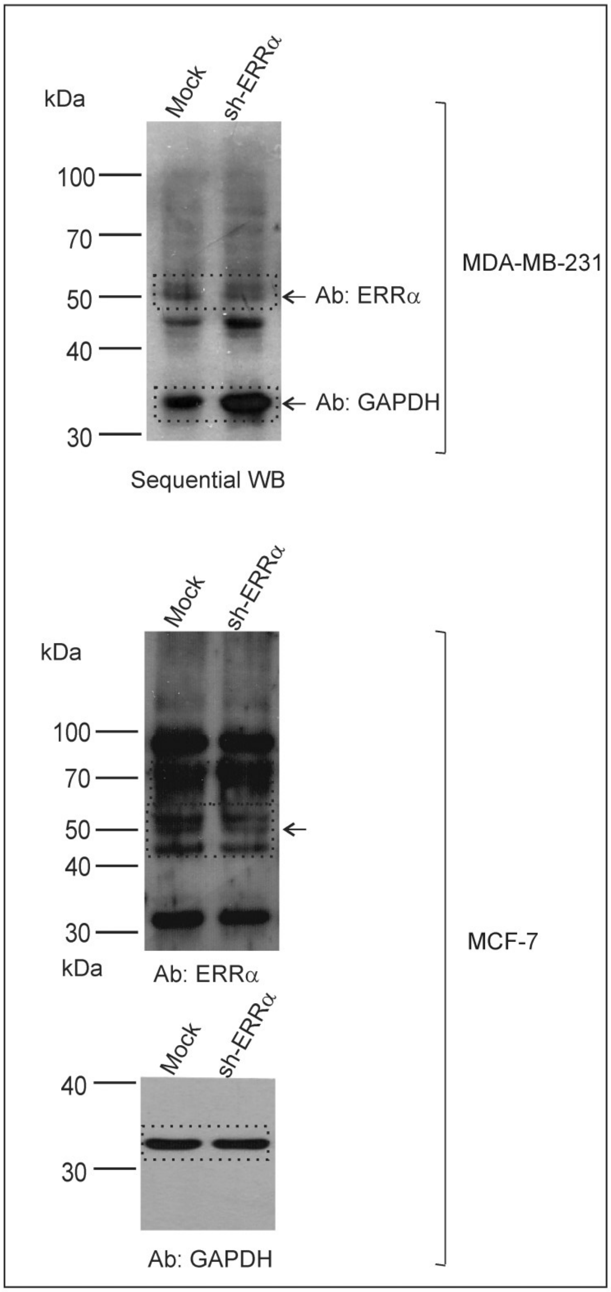

Figure 6 c

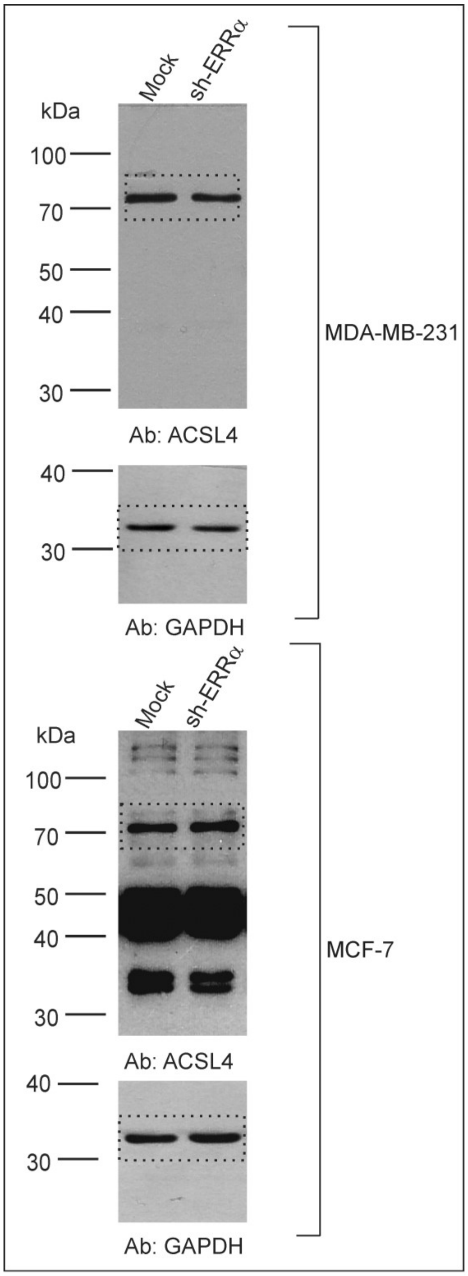

Figure 6d

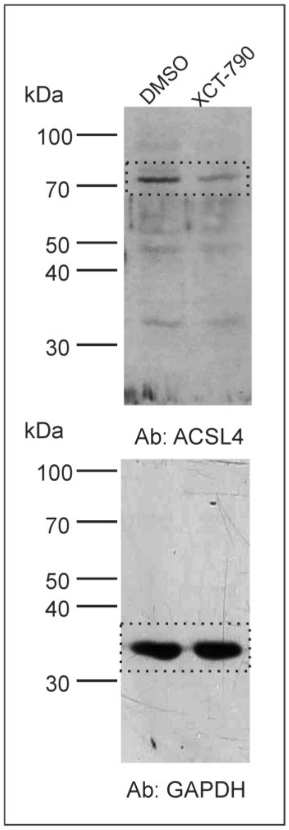

Figure 6g

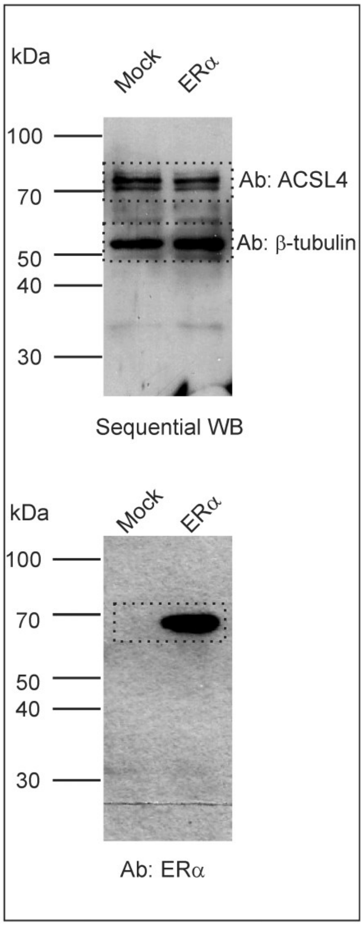

Figure 7c

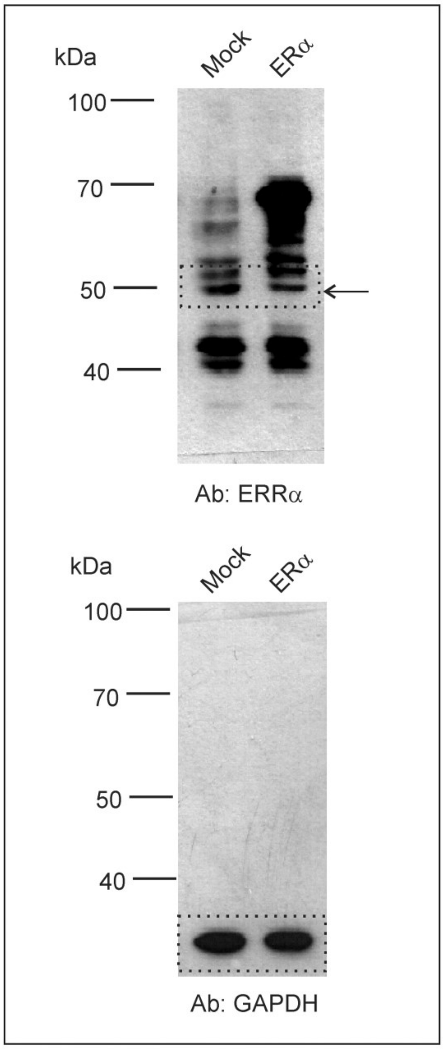
kDa
Mock
ERα
100
70
50
40
Ab: ERRα
kDa
Mock
ERα
100
70
50
40
Ab: GAPDH

Figure 7g
